# Difficulties and Countermeasures in Hospital Emergency Management for Fast-Lane Treatment of Acute Stroke During the COVID-19 Epidemic Prevention and Control

**DOI:** 10.3389/fneur.2020.604907

**Published:** 2020-11-27

**Authors:** Haojun Yang, Zhuohui Chen, Yishu Fan, Lan Xiang, Xinhang Hu, Tong Wu, Bo Xiao, Li Feng, Zhihong Zhao, Yunfang Chi, Mengqi Zhang

**Affiliations:** ^1^Department of Neurology, Xiangya Hospital of Central South University, Changsha, China; ^2^Department of Neurology, Hunan Provincial People's Hospital, Hunan Normal University, Changsha, China; ^3^Laizhou People's Hospital, Yantai, China

**Keywords:** coronavirus disease 2019, epidemic control, emergency guarantee for acute stroke, medicine popularization, medical education

## Abstract

**Background:** Coronavirus disease 2019 (COVID-19) has a long incubation period and a high degree of infectivity. Patients may not show specific signs or symptoms of upper respiratory tract infection, and the age of onset is similar to that of stroke. Furthermore, an increase in neurological conditions, specifically acute cerebrovascular disease, has been detected. Providing emergency treatment for acute stroke in accordance with the strict epidemic control measures is currently one of the main challenges, as acute stroke is rapid onset and a major cause of death and disability globally. We aimed to evaluate the emergency treatment system for acute stroke during the epidemic control period to provide a reference and basis for informing government and medical institutions on improving patient treatment rates during this period.

**Methods:** Difficulties faced in providing emergency treatment for stroke during an epidemic were investigated and combined with medical educational resources and clinical management experiences to construct an emergency treatment framework for acute stroke during the epidemic.

**Findings:** Currently, emergency treatment measures for acute stroke during the epidemic control period are limited because the main focus is on identifying COVID-19 comorbidities during the critical period. Establishing standards for patients in the neurological outpatient consultation rooms and emergency observation and resuscitation zones; implementing a fast-lane system for the emergency treatment of patients with acute stroke, and strengthening ward management and medicine popularization, can improve the treatment efficiency for stroke patients during the epidemic and provide a reference for peers in clinical practice.

**Interpretation:** Emergency treatment for acute stroke during COVID-19 epidemic control period requires a joint promotion of clinical, popularization, and teaching resources.

## Introduction

In December 2019, an outbreak of a novel coronavirus pneumonia (NCP) occurred in Wuhan, Hubei province, China, and it rapidly spread to different provinces in China, as well as countries in Southeast Asia, Europe, North America, and many others (1). The Chinese National Health Commission has listed NCP as a category B infectious disease, and the management measures for category A infectious diseases were adopted, indicating that new discovered cases are required to be reported within 2 and 6 h in the city and countryside, respectively (2, 3). According to the World Health Organization (WHO), the official name of the disease is coronavirus disease 2019 (COVID-19) and according to the International Committee on Taxonomy of Viruses, the virus is called severe acute respiratory syndrome coronavirus 2 (SARS-CoV-2) (4, 5). The main clinical presentations of COVID-19 were fever, fatigue, dry cough, and other respiratory symptoms (6–8). Although the main clinical manifestations are respiratory, an increase in neurological conditions, specifically acute cerebrovascular disease, has been detected (9–11). Stroke refers to acute neurological dysfunction due to a vascular cause, which tends to occur mostly in winter and spring and is rapid onset and a major cause of death and disability globally (12, 13). People with underlying diseases such as diabetes, hypertension and cardiovascular disease, were more associated with stroke (14–16). COVID-19 patients with underlying diseases had poorer prognosis and higher mortality rates (17, 18). Identifying COVID-19 comorbidities in a patient during the critical period in acute stroke treatment is a key point and a challenge in stroke management. Herein, we evaluate and describe not only the challenges in providing emergency treatment for acute stroke but also the possible response strategies to ensure efficient operation regarding acute stroke diagnosis and treatment during the COVID-19 epidemic and provide basic ideas for clinical practice.

## Challenges in Providing Emergency Treatment For Acute Stroke During the COVID-19 Epidemic Control Period

SARS-CoV-2 is an enveloped, positive-sense, single-stranded RNA virus belonging to the Betacoronavirus genus, and its sequence is highly homologous with that of SARS-like coronaviruses in bats (19–21). The main transmission routes include infected respiratory droplets and direct contact with the infected individual. Transmission through other routes such as aerosols and fecal–oral routes is still unclear and requires validation through further studies (22, 23). Humans are generally susceptible to the disease. Compared with SARS, SARS-CoV-2 has a longer incubation period and a high degree of infectivity. Patients may not show specific upper respiratory tract signs and symptoms (24). Therefore, COVID-19 tends to cause nosocomial infection and cross-infection, which poses a great challenge in epidemic control. The construction of an emergency treatment framework and strategy is an important prerequisite for the standardization of the diagnosis and treatment process and thus improving patient treatment efficiency, thereby having direct and key effects on improving treatment rate, thereby reducing mortality rate, improving public confidence, and alleviating social panic.

### Identification of COVID-19 Comorbidities During the Critical Period of Acute Stroke Treatment

Stroke, or cerebrovascular accident, involves injury to the central nervous system due to a vascular cause, which is a major cause of death and disability globally (24). Patients with COVID-19 alongside an underlying disease had poorer prognosis and higher mortality rates: the mortality rates of patients with diabetes, hypertension, and heart disease were 5.3, 2.8, and 4.2%, respectively (25). In addition to smoking, obesity, excessive alcohol consumption, and lack of exercise, hypertension and diabetes are the most important risk factors for stroke (26–28). A recent study showed ~81% of COVID-19 patients with ischemic stroke had known vascular risk factors, the commonest being hypertension (75%) followed by diabetes (50%), coronary disease or atrial fibrillation (29). The age at onset of COVID-19 is similar to that of stroke, and all underlying comorbidities are also important risk factors for stroke. Studies have shown the possibilities of cerebrovascular diseases being the initial symptoms of COVID-19, associating with a poor prognosis. The mortality rate of COVID-19 patients with stroke was higher than that previously reported in either COVID-19 respiratory infection alone, or acute ischemic stroke alone (30). Additionally, racial disparities in COVID-19 case counts and outcomes have been highlighted, especially among African American populations (30, 31). Underlying biological, genetic, or epigenetic characteristics along with less access to healthcare, and social and economic disparities might predispose to health differences and outcomes (32, 33). 84.6% of strokes were cortical and more than 50% of patients had no identifiable source, which were categorized as embolic stroke of unknown source (ESUS) (34). The common large vessel disease with ESUS indicates an increased risk of coagulopathy and endothelial dysfunction. Several COVID-19 series pointed infarcts typically followed a subcortical or distal cortical distribution and some mechanisms for stroke, which might be associated with the infection and its complications, including either the causation of acute cardiac injury, creation of antiphospholipid antibodies or even severe hypercoagulable conditions caused by D-dimer or fibrinogen abnormalities (9, 35, 36). Consistent with the previous studies, a high rate inflammatory markers was found in stroke population, such as the neutrophil-lymphocyte ratio, ferritin, D-dimer, procalcitonin, C-reactive protein (CRP) and lactate dehydrogenase (29, 37). Therefore, attention must be paid closely to the risk of comorbidities in COVID-19 during stroke treatment. Regarding the treatment of patients with acute stroke, time is of the essence. Identifying COVID-19 comorbidities in patients with acute stroke during the critical period and reducing delays caused by COVID-19 screening is the main challenges in the emergency treatment.

### Strong Popular Science Requirements for Acute Stroke

New media and we media have continuously emerged and become important sources of information for the public, which not only means that the media provides increasing popular medical knowledge, but also prompts the misinformation and rumors (38). Previous studies have shown that new media use and more media engagement was associated with negative psychological outcomes, while certain media content such as viewing heroic acts, speeches from experts, and knowledge of the disease and prevention was associated with positive psychological impact (39, 40). These studies highlighted the need for timely public health communication from official sources and debunking misinformation associated with the COVID-19 in real time by local communities and governments (41). The outline of “Healthy China 2030” highlighted that prevention was better than cure in the establishment of a healthy China and encouraged the strengthening of popular medical education and improving the public health communication (42, 43). Season type, especially winter and spring, and unhealthy lifestyles increase the risk for stroke (12, 13). How to prevent stroke at home, how to identify stroke, and the subsequent admission and treatment processes in hospitals during an epidemic are the main aspects of popular science. The deep learning of the admission and treatment processes in hospitals for patients' family members during the epidemic will not only avoid losing time due to unfamiliarity, but also strengthen their trust in the medical staff and reduce medical disputes which occur during COVID-19 screening (44, 45). However, the impact of COVID-19 on improving the doctor-patient relationship in China is still very controversial. Although the occupancy in most inpatient and outpatient clinics were reduced, the amount of work was increased by fundamental transitions of work flows, communication, staff structure and hygiene measures to cover the needs of prevention, treatment and follow-up care as well as protection of staff (46). Additionally, self-reported rates of anxiety symptoms and depression symptoms were high among medical staff under the outbreak of COVID-19 (47, 48). Therefore, how to balance the work and popular science of medical staff is another problem. How to rationally utilize the professional knowledge of medical students, combine teaching resources and medical popularization, cultivate new medical talents, and examine sustainable development mechanisms for combining social mission and the literacy training of medical students, science popularization, and public interests are problems that require crucial analytical investigation (49).

## Construction of an Acute Stroke Emergency Treatment Framework Under Epidemic Control Conditions

In this study, the problem of reducing the delay caused by COVID-19 screening faced during the treatment of patients with acute stroke under COVID-19 epidemic control conditions was used to propose a basic framework for fast lane treatment of acute haemorrhagic stroke and acute ischemic stroke. In this framework, the construction of a rational organizational framework is the basis for ensuring the rapid diagnosis and treatment of patients with acute stroke on the basis of COVID-19 control. The coordinated and orderly collaboration between various departments is a prerequisite for achieving resuscitation during the golden hour. Real-time standby of different teams provides important support for the emergency treatment of acute stroke. This strategy with a good response has been applied to hospitals in Changsha, China, aiming at providing a reference and basis for informing government and medical institutions on improving patient treatment rates during this period.

### Strengthening Staff Control and Standardizing Admission Screening

During the treatment of patients with acute stroke, attention should be paid to the risk of COVID-19 comorbidities, which is determined by the following criteria:

Epidemiological history: ① History of travel or residence to high-risk areas within 14 days before disease onset in patients with acute stroke; ② History of contact with people from the high-risk areas within 14 days before disease onset in patients with acute stroke; ③ Presence of disease clusters; ④ Close contact with patients with fever, fatigue, or respiratory symptoms

Clinical presentation: ① Fever or respiratory symptoms including weak and dry cough; ② Classical imaging presentation of COVID-19; ③ Normal or reduced white blood cell count or lymphocytopenia during the early stages of the disease.

#### Patient Admission Management Process in the Neurological Outpatient Consultation Room

**Figure 1:**
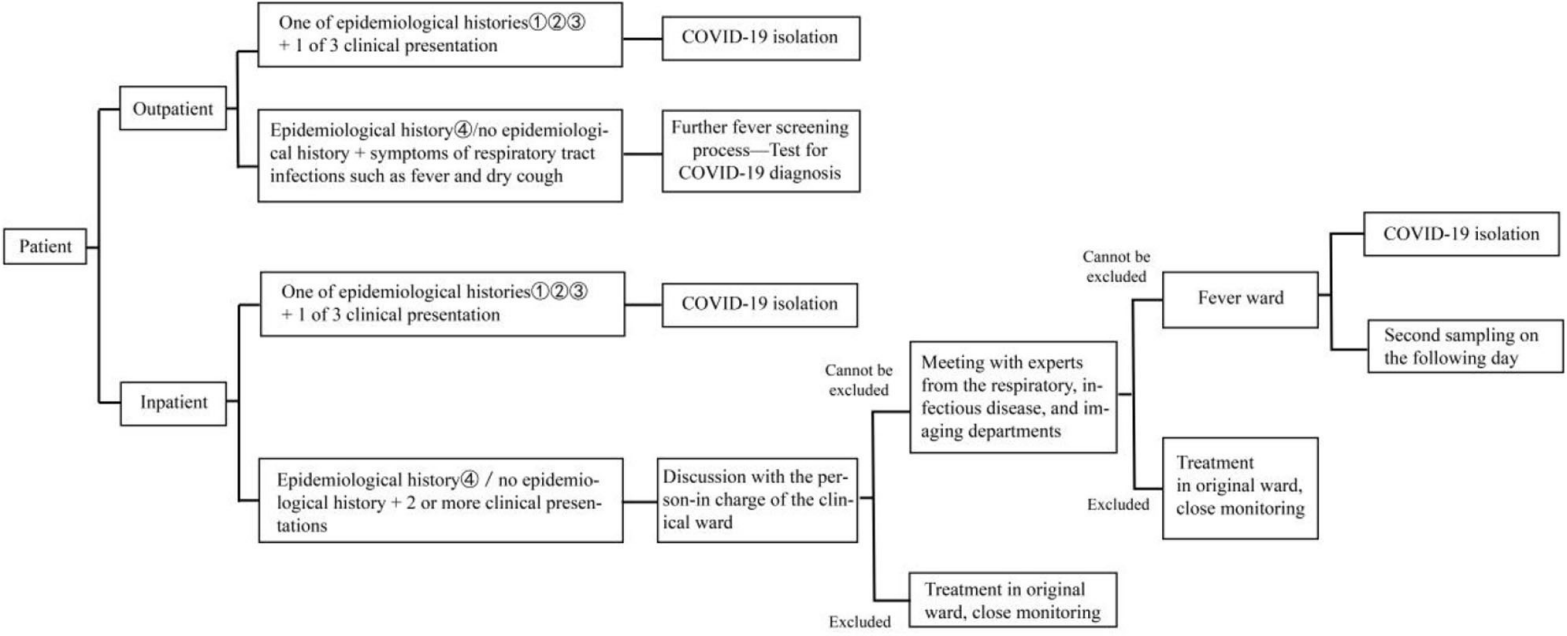
Neurology emergency department and inpatient screening process.

① Inpatient treatment should be avoided as much as possible for non-emergency outpatients, and elective admission and treatment should be carried out after the epidemic has been controlled.
② For outpatients who require hospitalization, temperature monitoring and detailed inquiry, on epidemiological contact history and clinical presentation for COVID-19 should be carried out. Lung computed tomography (CT), routine blood tests, erythrocyte sedimentation, CRP, procalcitonin, four-item pre-transfusion screening (HBsAg, HCV-Ab, TP-Ab, and HIV-Ab), and electrolyte tests should be carried out.
③ The above medical tests and relevant results are used to determine a suspected case of COVID-19 and whether the patient is admitted or entered the neurology emergency department for stroke and inpatient screening process (Figure 1).
④ For those who require inpatient treatment, the outpatient physician is responsible for notifying the chief resident physician in the ward.

#### Patient Admission Management Process in the Neurological Emergency Observation and Resuscitation Zones

The pre-screening and triage procedures are strictly complied with scientific screening and rational triaging of patients. Patients who visit the new medical emergency observation or resuscitation zones after pre-screening and triage at the emergency department will be hospitalized after specialist consultation according to the routine procedures, if they do not have a fever, epidemiological history, or respiratory symptoms. If fever and respiratory symptoms are present, ① Lung CT, blood routine, ordinary CRP, erythrocyte sedimentation, procalcitonin, four-item pre-transfusion, and electrolyte tests must be performed. ② If significant imaging presentation is absent, two emergency department team members will sign and confirm, and the comments of the imaging expert will be recorded (valid within 24 h of signing). No further screening is required, and the patient can be admitted by a specialist attending physician. ③ If COVID-19 cannot be excluded according to the consultation opinions of the emergency department expert group (including imaging), the patient will be transferred to a designated fever isolation zone in the hospital for further screening. On the following day, two pharyngeal swabs will be collected for SARS-CoV-2 nucleic acid screening. ④ Patients who are positive for SARS-CoV-2 will be transferred to designated hospitals, while those who are negative will undergo consultation by the emergency department expert group (including imaging). After COVID-19 exclusion, emergency department team members will sign. Simultaneously, the comments of the imaging expert will be documented in the medical records, and the patient will be admitted to the specialist department.

#### Fast Lane Treatment of Emergency Department for Acute Stroke

**Figure 2:**
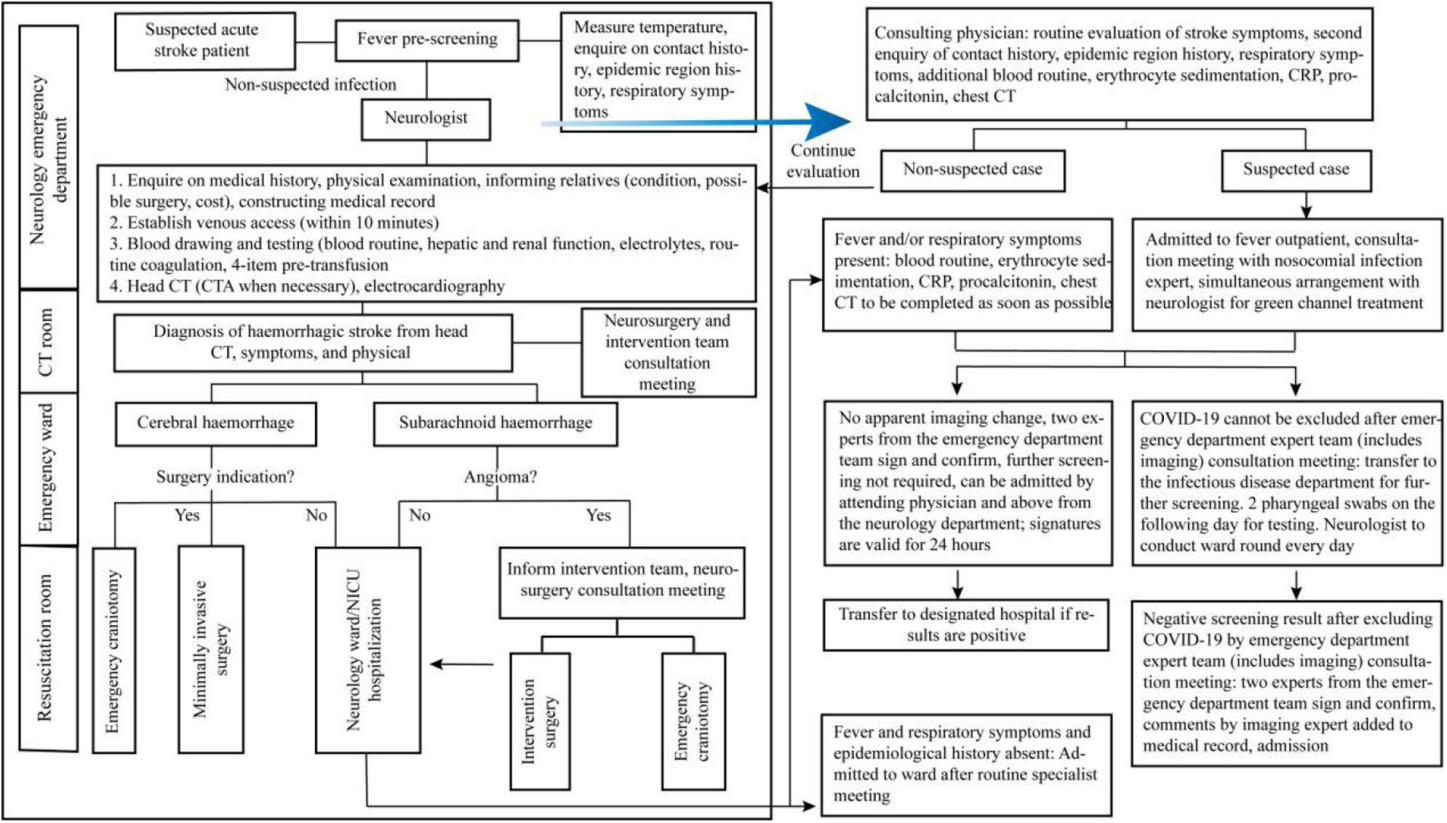
Acute haemorrhagic stroke fast lane management procedure during the COVID-19 epidemic.

**Figure 3:**
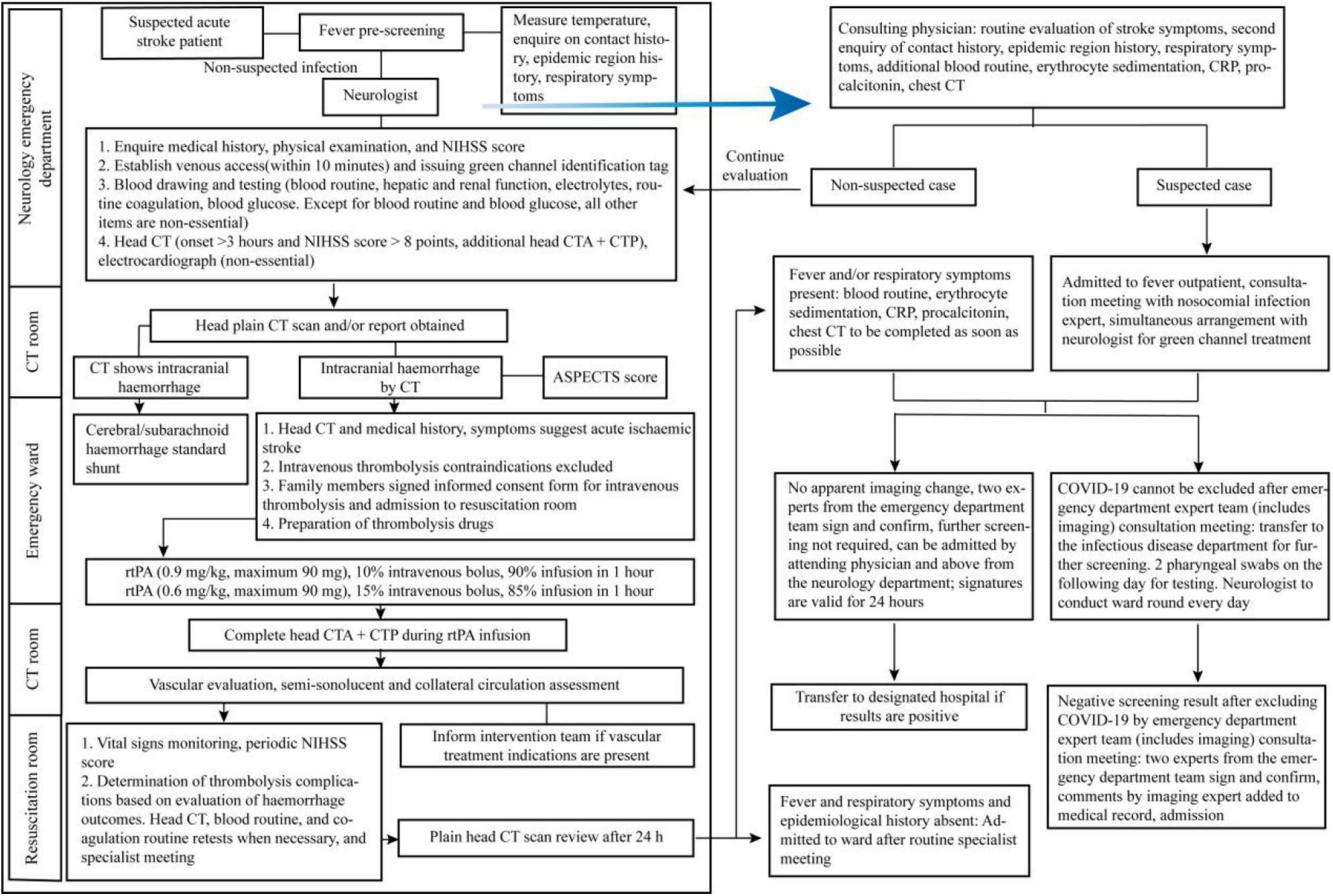
Acute ischemic stroke fast lane management procedure during the COVID-19 epidemic.

Due to the differences in resuscitation measures during an acute haemorrhagic stroke and acute ischemic stroke, we describe the fast lane procedures for acute haemorrhagic stroke (Figure 2) and acute ischemic stroke (Figure 3) during a COVID-19 epidemic and propose the following recommendations for the consultation of patients with acute stroke in the emergency department:

① For patients with suspected or confirmed COVID-19 along with comorbid critical cerebrovascular disorder, if the disease onset occurs outside of the hospital, then they should be transferred to the nearest National Health Commission-designated hospital for treatment. Patients with acute cerebrovascular disorder who seek medical attention at our hospital should undergo scientific screening, rational triage, and timely quarantine in strict accordance with the COVID-19 emergency department pre-screening and triage procedures.
② For suspected patients who need to enter the fast lane of stroke, dedicated medical staff will accompany them to the COVID-19 screening zone, and screening will be carried out according to the fever outpatient procedure. Simultaneously, neurologists will be assigned to assist in the fast lane treatment of stroke.
③ Aggressive thrombolysis treatment should be administered simultaneously with the screening for acute ischemic stroke in patients who fulfill the criteria for a suspected COVID-19 case, within the time window for intravenous thrombolysis and if there is no contraindication for intravenous thrombolysis (50). Endovascular recanalization therapy should also be considered in suspected COVID-19 patients with acute ischemic stroke due to large artery occlusion within 6 h [or within 24 h in some cases (51)] after onset (52). Stroke team members must be careful of COVID-19 exposure during clinical evaluation and performance of imaging and laboratory procedures of stroke patients with suspected or confirmed COVID-19 infection, especially in the process of intravenous thrombolysis and mechanical thrombectomy (53). For patients with severe/critical COVID-19, the pros and cons should be weighed. In principle, pneumonia is treated first. After completing the treatment, the patient is transferred to the isolation room, and neurologists are organized to conduct ward rounds in the isolation room daily.
④ As patients with cerebral hemorrhage or subarachnoid hemorrhage often present with fever, the procedure must be strictly followed for identification and screening. Patients suspected to have COVID-19 will not enter the catheterisation room for angiography, craniotomy, or intervention treatment for the disease. These patients should be isolated and treated with routine medical conservative treatment. Time selecting operations will be performed when COVID-19 is ruled out.

### Strengthening Ward Management

#### Ward Control and Management

① A dedicated entrance is set up at the ordinary neurological ward for demographic information registration and temperature measurement by the staff, and it restricts access to unauthorized people. Only one caregiver can accompany every patient. In the neurological ICU ward, a telephone is used to communicate with the staff about the patient's condition. When necessary, visitation is carried out. Workers will use their staff cards, inpatients will use their wrist bands, and caregivers will use their caregiver card for access. People without their facial masks on are not allowed into the medical zone.
② Clean zones, potential contamination zones, and contamination zones are designated in the neurological ordinary and ICU wards; staff paths are set up; and bedside diagnosis, treatment, and nursing are advocated.
③ Emergency isolation wards are set up in the ward, which are equipped with sufficient disinfection and protective equipment to respond to acute respiratory infection for quarantine and treatment of suspected and confirmed patients. Work is carried out according to relevant systems and procedures to achieve isolation, disinfection, and protection requirements stipulated in the relevant guidelines.
④ The neurological ordinary and ICU wards will report data on fever in inpatients from the midnight of the previous day to the present midnight to the medical affairs department at 8 a.m. daily. The infectious disease management office of the medical affairs department will complete the summary at 8:30 a.m. daily and submit the report.
⑤ If suspected or confirmed patients are discovered in the ward, relevant emergency plans and work procedures will be activated rapidly. The suspected or confirmed patients are transferred to the emergency isolation wards [point ③ ], who need treatment and referral according to the requirements later. The entire ward needs to be thoroughly disinfected, and both medical staff, patients and caregivers in the ward are classified as close contacts. Medical staff and caregivers should be isolated immediately and patients should be observed and isolated while treating. Another group of medical staff will take over this ward with protection measures.
⑥ Specialist diagnosis and treatment or care of suspected or confirmed patients are carried out to ensure that medical staff achieve the corresponding protection grade in the relevant regulations. Additionally, access of non-medical staff is restricted, and visitations are not allowed according to the principle.
⑦ After patients are transferred out, the contact environment is treated according to the medical institution disinfection technical specifications (54, 55).

#### Medical Staff Management

##### Entire departmental staff training

Training on COVID-19 case discovery and reporting, epidemiological survey, sample collection, laboratory testing, medical treatment and nosocomial infection control, and personal protection will be given to all staff members in the department to make them familiar with COVID-19 control knowledge, methods, and techniques; understand laws and obligations related to epidemic control; and achieve early identification, early reporting, early quarantine, early diagnosis, and early treatment.

##### Overall deployment of medical resources

Overall deployment of medical resources in the department, rational establishment of medical echelons, rational scheduling, and organization of a preparatory echelon are carried out. Graded protection is carried out according to the position and zone protection standards and material allocation requirements. Department staff are supervised to ensure that they strictly comply with the medical staff protective gear gowning/de-gowning procedure (56), and suspected medical staff are quarantined and treated in a timely manner. The protection measures for different roles are as follows:

① Primary protection: (a) Diagnosis, treatment and nursing of ordinary inpatients; (b) Triaging, and registration of outpatients in the fever outpatient clinic and timely data reporting; (c) Cleaning and disinfection of ordinary zones; (d) Collection of medical waste from ordinary patients; (e) Ordinary cleaning work; (f) Testing ordinary patients by medical technicians; (g) While processing the samples from suspected patients in the laboratory, it is recommended that the staff wear masks (N95), protective goggles (anti-fog) or protective face shields for primary protection.
② Secondary protection: (a) Diagnosis and treatment, care, and processing medical waste from infected or suspected patients; (b) Collection of medical waste from infected or suspected patients; (c) Cleaning areas that have been exposed to patients with suspected or confirmed COVID-19.
③ Tertiary protection: Diagnosis and treatment, care, and surgical operation in patients with suspected severe infections (such as tracheotomy or intubation).

##### Concern for the physical and mental health of medical staff

During the COVID-19 epidemic, clinical staff will face physical, intellectual, and psychological challenges, and protecting them is key to eradicating the epidemic (57, 58). Therefore, rational arrangement of manpower resources and shifts should be carried out to avoid over-exhaustion among the medical staff. Proactive health monitoring should be carried out based on the characteristics of different positions and risk assessment, and several measures should be employed to ensure good physical and mental health among the medical staff as well as the families of clinical frontline staff. If medical staff develop psychological problems and require psychological counseling and drug intervention, a psychiatrist should be asked to assist in psychological intervention when necessary.

#### Inpatient Management

If a suspected COVID-19 case is identified in a ward, then the patient should be quarantined in a single room, and activities and visits from family members should be restricted. Medical staff should wear protective gears (N95 mask and disposable isolation gown for primary protection), avoid contact with patients as much as possible, and avoid repeated movement in and out the ward. Emergency chest CT and routine blood tests, ordinary CRP, erythrocyte sedimentation, procalcitonin, four-item pre-transfusion, and electrolyte tests should be performed. The person in charge of the ward will examine and identify patients. For specific procedure, please refer to “neurological emergency outpatient and inpatient screening process (Figure 1).” Close attention must be paid to the following points during the management of inpatients:

① Quarantine should be carried out early for suspected or confirmed patients.
② Inpatients should be guided on how to correctly select and wear masks, correct cough etiquette, and hand hygiene.
③ Strengthening the management of patient visits by caregivers.
④ Proactive advocacy on epidemic control knowledge in inpatients and caregivers.

#### Medical Waste Management and Treatment

**Figure 4:**
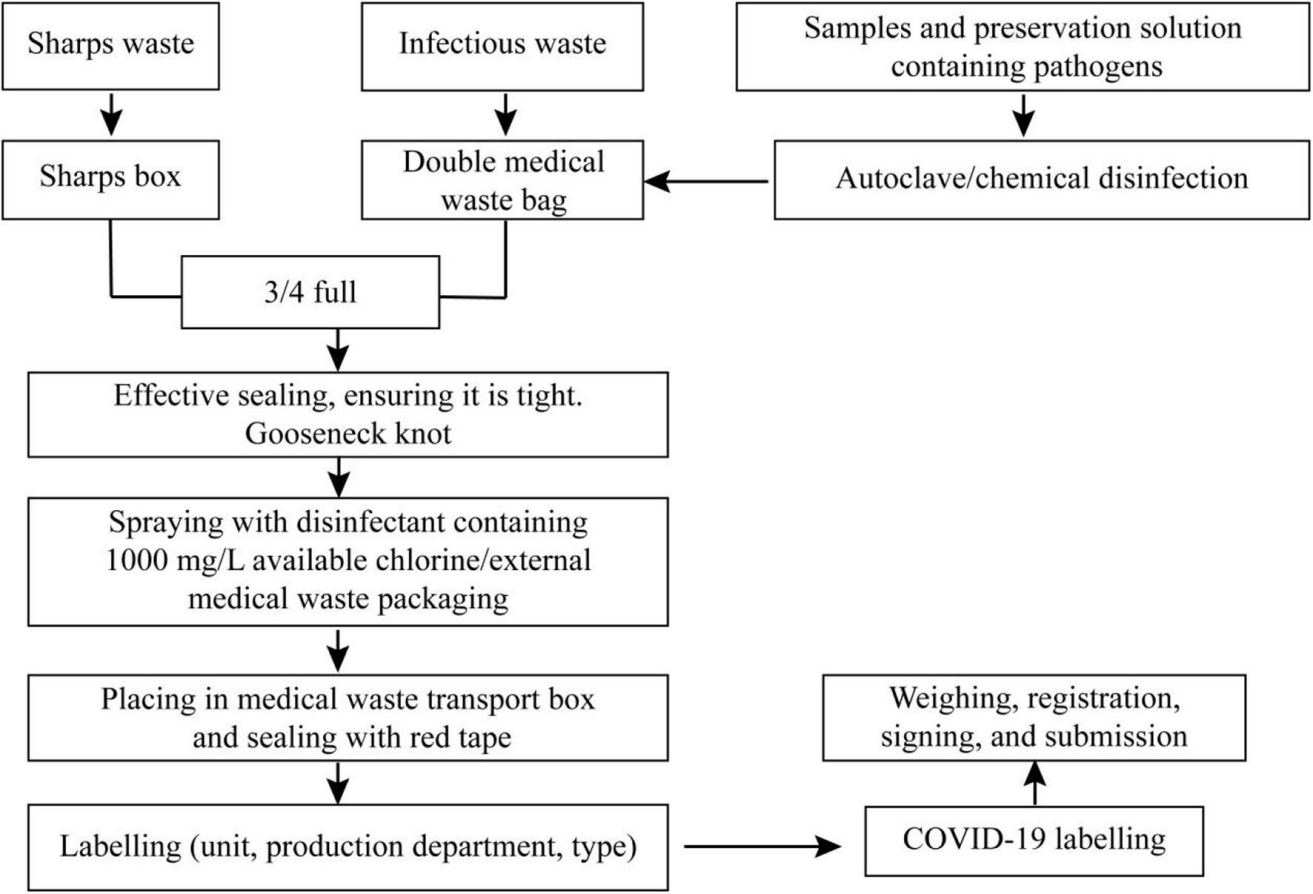
Medical waste management and treatment flowchart.

Different medical waste (sharps waste, infectious waste, samples, and preservation solution containing pathogens, etc.) should be collected in order (Figure 4). Tightness, cleanliness, color specificity, labeling, and processing registration are the most important points in the management and treatment of medical waste.

## Construction of an Acute Stroke Emergency Treatment Team During Epidemic Control

With the continuous emergence of new media, people are no longer restricted to television and newspapers to acquire information, as more information is acquired from various new media platforms (59–61). However, this increases misinformation and rumors. When faced with a public health emergency such as COVID-19, the public tended to be misinformed on the Internet when they were unable to obtain timely, authoritative, and scientific information (62, 63), which resulted in panic buying of medical supplies, increased nervousness in the public and caused severe negative social effects. This showed the importance of science popularization and the emergency treatment team. In this study, acute stroke was used as a starting point to construct science popularization and an emergency treatment team during an epidemic. First, the selection of science popularization content should include the following principles: ① Determine the target population; ② Target the current status of the epidemic; ③ Conform to actual situation; ④ Get accurate and easily understandable content. Therefore, science popularization was divided into COVID-19 epidemic and stroke modules to describe the disease characteristics, type of onset, and prevention. The connections between the contents are ensured, and long articles were avoided. This required professional knowledge and language summarisation ability of the popular science author. In addition to text, animations, comics, and videos could be combined in many ways to make popular science content more interesting and acceptable to the public. A three-layered structure is adopted for the construction of a science popularization team: ① Editorial team: consisted mainly of medical professionals with a Ph.D. degree or students with a master's or an undergraduate degree, who were responsible for selecting and compiling popular science content and submitting manuscripts to the review group. ② Review team: mainly consisted of hospital professors and physicians, who were responsible for reviewing manuscripts. ③ Publicity team: consisted of mostly the hospital publicity party committee, who were responsible for the publication of the final popular science manuscript on various major platforms such as new media platforms and WeChat accounts and for making short public interest clips and uploading to TikTok and other self-media platforms.

## Summary

During the COVID-19 epidemic, identifying patient with comorbid COVID-19 symptoms during the golden hour for acute stroke treatment is a challenge in stroke control. This requires an action framework and a standardized procedure. Rationally utilizing the professional knowledge of medical students and combining teaching resources will not only improve the medical knowledge of the public and reduce frontline stress in the clinical practice but also be significant in fighting against the epidemic. Moreover, promoting medical education reforms on the Internet is highly influential and should be further considered.

## Author Contributions

MZ conceptualized the study, acquired funding, and administered the project. HY and MZ wrote the original draft. ZC and YF provided the resources. LX, XH, TW, ZZ, and LF worked on validation. BX supervised the study. MZ, HY, and YC reviewed and edited the manuscript.

## Conflict of Interest

The authors declare that the research was conducted in the absence of any commercial or financial relationships that could be construed as a potential conflict of interest.

## References

[1] Cucinotta D Vanelli M WHO declares COVID−19 a pandemic. Acta Biomed. (2020) 91:157–60. 10.23750/abm.v91i1.9397 32191675 PMC7569573

[2] Yang H Chi Y Chen Z Fan Y Wu H Hu X Differential diagnosis and hospital emergency management for fastlane treatment of central nervous system infection under the COVID−19 epidemic in Changsha, China. Front. Neurol. (2020) 11:555202 10.3389/fneur.2020.555202 33192989 PMC7606862

[3] Society of Pathological Doctors Chinese Medical Doctors Association Chinese Society of Pathology Chinese Medical Association Provisional guidelines on autopsy practice for deaths associated with COVID−19. Zhonghua Bing Li Xue Za Zhi. (2020) 49:406–10. 32153166 10.3760/cma.j.cn112151-20200309-00184

[4] Zhang T Wu Q Zhang Z Probable pangolin origin of SARS–CoV−2 associated with the COVID−19 outbreak. Curr. Biol. (2020) 30:1346–51.e2. 10.1016/j.cub.2020.03.022 32197085 PMC7156161

[5] Coronaviridae Study Group of the International Committee on Taxonomy of Viruses. The species severe acute respiratory syndrome–related coronavirus: classifying 2019-nCoV and naming it SARS–CoV−2. Nat. Microbiol. (2020) 5:536–44. 10.1038/s41564-020-0695-z 32123347 PMC7095448

[6] Xu XW Wu XX Jiang XG Xu KJ Ying LJ Ma CL Clinical findings in a group of patients infected with the 2019 novel coronavirus (SARS–Cov−2) outside of Wuhan, China: retrospective case series. BMJ. (2020) 368:m606 10.1136/bmj.m606 32075786 PMC7224340

[7] Chen N Zhou M Dong X Qu J Gong F Han Y. Epidemiological and clinical characteristics of 99 cases of 2019 novel coronavirus pneumonia in Wuhan, China: a descriptive study. Lancet. (2020) 395:507–13. 10.1016/S0140-6736(20)30211-7 32007143 PMC7135076

[8] Yao Y Cao J Wang Q Shi Q Liu K Luo Z. D–dimer as a biomarker for disease severity and mortality in COVID−19 patients: a case control study. J. Intensive Care. (2020) 8:49. 10.1186/s40560-020-00466-z 32665858 PMC7348129

[9] Mao L Jin H Wang M Hu Y Chen S He Q. Neurologic manifestations of hospitalized patients with coronavirus disease 2019 in Wuhan, China. JAMA Neurol. (2020) 77:683–90. 10.1001/jamaneurol.2020.1127 32275288 PMC7149362

[10] Hernández–Fernández F Valencia HS Barbella-Aponte RA Collado-Jiménez R Ayo-Martín Ó Barrena C. Cerebrovascular disease in patients with COVID−19: neuroimaging, histological and clinical description. Brain. (2020) 143:3089–103. 10.1093/brain/awaa239 32645151 PMC7454411

[11] Fifi JT Mocco J. COVID−19 related stroke in young individuals. Lancet Neurol. (2020) 19:713–15. 10.1016/S1474-4422(20)30272-6 32822622 PMC7434432

[12] Liao JN Chao TF Liu CJ Chen SJ Hung CL Lin YJ. Seasonal variation in the risk of ischemic stroke in patients with atrial fibrillation: a nationwide cohort study. Heart Rhythm. (2018) 15:1611–6. 10.1016/j.hrthm.2018.06.043 29969675

[13] Turin TC Kita Y Murakami Y Rumana N Sugihara H Morita Y. Higher stroke incidence in the spring season regardless of conventional risk factors: Takashima Stroke Registry, Japan, 1988–2001. Stroke. (2008) 39:745–52. 10.1161/STROKEAHA.107.495929 18258821

[14] Cho SK Sohn J Cho J Noh J Ha KH Choi YJ. Effect of socioeconomic status and underlying disease on the association between ambient temperature and ischemic stroke. Yonsei Med. J. (2018) 59:686–92. 10.3349/ymj.2018.59.5.686 29869467 PMC5990672

[15] Chen R Ovbiagele B Feng W. Diabetes and stroke: epidemiology, pathophysiology, pharmaceuticals and outcomes. Am. J. Med. Sci. (2016) 351:380–6. 10.1016/j.amjms.2016.01.011 27079344 PMC5298897

[16] Cipolla MJ Liebeskind DS Chan SL. The importance of comorbidities in ischemic stroke: Impact of hypertension on the cerebral circulation. J. Cereb. Blood Flow Metab. (2018) 38:2129–49. 10.1177/0271678X18800589 30198826 PMC6282213

[17] Adhikari SP Meng S Wu YJ Mao YP Ye RX Wang QZ. Epidemiology, causes, clinical manifestation and diagnosis, prevention and control of coronavirus disease (COVID−19) during the early outbreak period: a scoping review. Infect. Dis. Poverty. (2020) 9:29. 10.1186/s40249-020-00646-x 32183901 PMC7079521

[18] Han R Huang L Jiang H Dong J Peng H Zhang D. Early clinical and CT manifestations of coronavirus disease 2019 (COVID−19) pneumonia. AJR Am. J. Roentgenol. (2020) 215:338–43. 10.2214/AJR.20.22961 32181672

[19] Glass WG Subbarao K Murphy B Murphy PM. Mechanisms of host defense following severe acute respiratory syndrome–coronavirus (SARS–CoV) pulmonary infection of mice. J. Immunol. (2004) 173:4030–9. 10.4049/jimmunol.173.6.4030 15356152

[20] Li Q Guan X Wu P Wang X Zhou L Tong Y. Early transmission dynamics in wuhan, china, of novel coronavirus–infected pneumonia. N. Engl. J. Med. (2020) 382:1199–1207. 10.1056/NEJMoa2001316 31995857 PMC7121484

[21] Giovanetti M Angeletti S Benvenuto D Ciccozzi M. A doubt of multiple introduction of SARS–CoV−2 in Italy: a preliminary overview. J. Med. Virol. (2020) 92:1634–36. 10.1002/jmv.25773 32190908 PMC7228217

[22] Yuen KS Ye ZW Fung SY Chan CP Jin DY. SARS–CoV−2 and COVID−19: the most important research questions. Cell Biosci. (2020) 10:40. 10.1186/s13578-020-00404-4 32190290 PMC7074995

[23] Lu X Zhang L Du H Zhang J Li YY Qu J SARS–CoV−2 infection in children. N. Engl. J. Med. (2020) 382:1663–5. 10.1056/NEJMc2005073 32187458 PMC7121177

[24] Campbell BCV Khatri P Stroke. Lancet. (2020) 396:129–142. 10.1016/S0140-6736(20)31179-X 32653056

[25] Deng SQ Peng HJ. Characteristics of and public health responses to the coronavirus disease 2019 outbreak in china. J. Clin. Med. (2020) 9:575. 10.3390/jcm9020575 32093211 PMC7074453

[26] Zhang M Tang M Wu Q Wang Z Chen Z Ding H. LncRNA DANCR attenuates brain microvascular endothelial cell damage induced by oxygen–glucose deprivation through regulating of miR−33a−5p/XBP1s. Aging. (2020) 12:1778–91. 10.18632/aging.102712 31986122 PMC7053632

[27] Zhang MQ Zhou L Deng QF Xie YY Xiao TQ Cao YZ. Ultra–high–resolution 3D digitalized imaging of the cerebral angioarchitecture in rats using synchrotron radiation. Sci. Rep. (2015) 5:14982. 10.1038/srep14982 26443231 PMC4595735

[28] Li HL Ding H Yin XZ Chen ZH Tang B Sun JY. Comparison of high–resolution synchrotron–radiation–based phase–contrast imaging and absorption–contrast imaging for evaluating microstructure of vascular networks in rat brain: from 2D to 3D views. J. Synchrotron. Radiat. (2019) 26(Pt 6):2024–32. 10.1107/S1600577519011688 31721747

[29] Tiwari A Berekashvili K Vulkanov V Agarwal S Khaneja A Turkel–Parella D. Etiologic subtypes of ischemic stroke in SARS–CoV−2 patients in a cohort of New York city hospitals. Front. Neurol. (2020) 11:1004. 10.3389/fneur.2020.01004 33041972 PMC7527497

[30] Dmytriw AA Phan K Schirmer C Settecase F Heran MKS Efendizade A. Ischaemic stroke associated with COVID-19 and racial outcome disparity in North America. J Neurol Neurosurg Psychiatry. (2020). 10.1136/jnnp-2020-324653. [Epub ahead of print]. 32801118

[31] Chowkwanyun M Reed AL Jr. Racial health disparities and Covid−19—caution and context. N. Engl. J. Med. (2020) 383:201–3. 10.1056/NEJMp2012910 32374952

[32] Yancy CW. COVID−19 and African Americans. JAMA. (2020) 323:1891–2. 10.1001/jama.2020.6548 32293639

[33] Vepa A Bae JP Ahmed F Pareek M Khunti K. COVID−19 and ethnicity: a novel pathophysiological role for inflammation. Diabetes Metab. Syndr. (2020) 14:1043–51. 10.1016/j.dsx.2020.06.056 32640416 PMC7326443

[34] Grewal P Pinna P Hall JP Dafer RM Tavarez T Pellack DR. Acute ischemic stroke and COVID−19: experience from a comprehensive stroke center in Midwest US. Front. Neurol. (2020) 11:910. 10.3389/fneur.2020.00910 32973666 PMC7468395

[35] Zhang Y Xiao M Zhang S Xia P Cao W Jiang W. Coagulopathy and antiphospholipid antibodies in patients with COVID−19. N. Engl. J. Med. (2020) 382:e38. 10.1056/NEJMc2007575 32268022 PMC7161262

[36] Helms J Kremer S Merdji H Clere–Jehl R Schenck M Kummerlen C Neurologic features in severe SARS–CoV−2 Infection. N. Engl. J. Med. (2020) 382:2268–70. 10.1056/NEJMc2008597 32294339 PMC7179967

[37] Beyrouti R Adams ME Benjamin L Cohen H Farmer SF Goh YY. Characteristics of ischaemic stroke associated with COVID−19. J. Neurol. Neurosurg. Psychiatry. (2020) 91:889–91. 10.1136/jnnp-2020-323586 32354768 PMC7231545

[38] González–Padilla DA Tortolero–Blanco L. Social media influence in the COVID−19 pandemic. Int. Braz. J. Urol. (2020) 46(Suppl. 1):120–4. 10.1590/s1677-5538.ibju.2020.s121 32550706 PMC7719982

[39] Chao M Xue D Liu T Yang H Hall BJ. Media use and acute psychological outcomes during COVID−19 outbreak in China. J. Anxiety Disord. (2020) 74:102248. 10.1016/j.janxdis.2020.102248 32505918 PMC7255752

[40] Depoux A Martin S Karafillakis E Preet R Wilder–Smith A Larson H. The pandemic of social media panic travels faster than the COVID−19 outbreak. J. Travel Med. (2020) 27:taaa031. 10.1093/jtm/taaa031 32125413 PMC7107516

[41] Islam MS Sarkar T Khan SH Mostofa Kamal AH Hasan SMM Kabir A COVID−19–related infodemic and its impact on public health: a global social media analysis. Am. J. Trop. Med. Hyg. (2020) 103:1621–9. 10.4269/ajtmh.20-0812 32783794 PMC7543839

[42] Song P Jin C Tang W. New medical education reform in China: towards healthy China 2030. Biosci. Trends. (2017) 11:366–9. 10.5582/bst.2017.01198 28904325

[43] Chen P Li F Harmer P. Healthy China 2030: moving from blueprint to action with a new focus on public health. Lancet Public Health. (2019) 4:e447. 10.1016/S2468-2667(19)30160-4 31493840

[44] Gao B Dong J. Does the impact of COVID−19 improve the doctor–patient relationship in China? Am. J. Med. Sci. (2020) 360:305–6. 10.1016/j.amjms.2020.05.039 32563570 PMC7831870

[45] Vallelonga F Elia F. Doctor–patient relationship at the time of COVID−19: travel notes. Intensive Care Med. (2020) 46:1802. 10.1007/s00134-020-06152-w 32548769 PMC7295932

[46] Härter M Bremer D Scherer M von dem Knesebeck O Koch–Gromus U. Impact of COVID−19–pandemic on clinical care, work flows and staff at a University Hospital: results of an interview–study at the UKE. Gesundheitswesen. (2020) 82:676–81. 10.1055/a-1226-6828 32823355 PMC7516358

[47] Liu Y Chen H Zhang N Wang X Fan Q Zhang Y. Anxiety and depression symptoms of medical staff under COVID−19 epidemic in China. J. Affect Disord. (2020) 278:144–8. 10.1016/j.jad.2020.09.004 32961409 PMC7475769

[48] Zhu J Sun L Zhang L Wang H Fan A Yang B. Prevalence and influencing factors of anxiety and depression symptoms in the first–line medical staff fighting against COVID−19 in Gansu. Front. Psychiatry. (2020) 11:386. 10.3389/fpsyt.2020.00386 32411034 PMC7202136

[49] The Lancet. The best science for achieving Healthy China 2030. Lancet. (2016) 388:1851. 10.1016/S0140-6736(16)31842-6 27751378

[50] Lin L Li TS Interpretation of “guidelines for the diagnosis and treatment of novel coronavirus (2019–nCoV) infection by the National Health Commission (Trial Version 5)”. Zhonghua Yi Xue Za Zhi. (2020) 100:805–7. 10.3760/cma.j.issn.0376-2491.2020.0001 32234150

[51] Ko SB Park HK Kim BM Heo JH Rha JH Kwon SU. 2019 Update of the Korean clinical practice guidelines of stroke for endovascular recanalization therapy in patients with acute ischemic stroke. J. Stroke. (2019) 21:231–40. 10.5853/jos.2019.00024 30991800 PMC6549067

[52] Zhao J Rudd A Liu R. Challenges and potential solutions of stroke care during the coronavirus disease 2019 (COVID−19) outbreak. Stroke. (2020) 51:1356–7. 10.1161/STROKEAHA.120.029701 32228369 PMC7219852

[53] Qureshi AI Abd–Allah F Al–Senani F Aytac E Borhani–Haghighi A Ciccone A. Management of acute ischemic stroke in patients with COVID−19 infection: report of an international panel. Int. J. Stroke. (2020) 15:540–54. 10.1177/1747493020923234 32362244

[54] Shi XM. The critical role of environmental health and disinfection in the prevention and control of COVID−19 pandemic. Zhonghua Yu Fang Yi Xue Za Zhi. (2020) 54:918–22. 10.3760/cma.j.cn112150-20200405-00516 32388938

[55] Hernández–Navarrete MJ Celorrio–Pascual JM Lapresta Moros C Solano Bernad VM. Principles of antisepsis, disinfection and sterilization. Enferm. Infecc. Microbiol. Clin. (2014) 32:681–8. 10.1016/j.eimc.2014.04.003 25023372

[56] Jin YH Zhan QY Peng ZY Ren XQ Yin XT Cai L. Chemoprophylaxis, diagnosis, treatments, and discharge management of COVID-19: an evidence-based clinical practice guideline (updated version). Mil Med Res. (2020) 7:41. 10.1186/s40779-020-00270-8 32887670 PMC7472403

[57] Huang J Liu F Teng Z Chen J Zhao J Wang X. Care for the psychological status of frontline medical staff fighting against COVID−19. Clin. Infect. Dis. (2020) 3:ciaa385. 10.1093/cid/ciaa385 32246142 PMC7184370

[58] Zhou Y Wang W Sun Y Qian W Liu Z Wang R. The prevalence and risk factors of psychological disturbances of frontline medical staff in china under the COVID−19 epidemic: workload should be concerned. J. Affect Disord. (2020) 277:510–4. 10.1016/j.jad.2020.08.059 32882508 PMC7448730

[59] Jabbar A Gasser RB Lodge J. Can new digital technologies support parasitology teaching and learning? Trends Parasitol. (2016) 32:522–30. 10.1016/j.pt.2016.04.004 27131629

[60] Lin DJ Cudkowicz ME Cho TA. Opinion and Special Articles: challenges and opportunities in defining career identity in academic neurology. Neurology. (2018) 91:670–2. 10.1212/WNL.0000000000006284 30275123

[61] Chan MM Barchino R Medina–Merodio JA de la Roca M Sagastume F. MOOCs, an innovative alternative to teach first aid and emergency treatment: a practical study. Nurse Educ. Today. (2019) 79:92–97. 10.1016/j.nedt.2019.05.008 31112846

[62] Gao J Zheng P Jia Y Chen H Mao Y Chen S. Mental health problems and social media exposure during COVID−19 outbreak. PLoS ONE. (2020) 15:e0231924. 10.1371/journal.pone.0231924 32298385 PMC7162477

[63] Ni MY Yang L Leung CMC Li N Yao XI Wang Y. Mental health, risk factors, and social media use during the COVID−19 epidemic and cordon sanitaire among the community and health professionals in Wuhan, China: cross–sectional survey. JMIR Ment. Health. (2020) 7:e19009. 10.2196/19009 32365044 PMC7219721

